# Volatolomics in Bacterial Ecotoxicology, A Novel Method for Detecting Signatures of Pesticide Exposure?

**DOI:** 10.3389/fmicb.2018.03113

**Published:** 2019-01-08

**Authors:** Kevin Hidalgo, Jeremy Ratel, Frederic Mercier, Benedicte Gauriat, Philippe Bouchard, Erwan Engel

**Affiliations:** ^1^INRA UR370 QuaPA, MASS Group, Saint-Genès-Champanelle, France; ^2^Thermo Fisher Scientific ZA de Courtaboeuf, Villebon-sur-Yvette, France; ^3^CNRS, Laboratoire Microorganismes: Genome et Environnement, Université Clermont Auvergne, Clermont-Ferrand, France

**Keywords:** VOC, marker of exposure, pesticides, microbial ecotoxicology, *Pseudomonas fluorescens*, *Bacillus megaterium*

## Abstract

Volatile organic compounds (VOC) produced by microorganisms in response to chemical stressor showed recently increasing attention, because of possible environmental applications. In this work, we aimed to bring the first proof of concept that volatolomic (i.e., VOCs analysis) can be used to determine candidate VOC markers of two soil bacteria strains (*Pseudomonas fluorescens* SG-1 and *Bacillus megaterium* Mes11) exposure to pesticides. VOC determination was based on solid-phase microextraction (SPME) coupled with gas chromatography-mass spectrometry (GC-MS). Accordingly, we highlighted a set of bacterial VOCs modulated in each strains according to the nature of the pesticide used. Three out these VOCs were specifically modulated in *P. fluorescens* SG-1 when exposed with two pyrethroid pesticides (deltamethrine and cypermethrine): 2-hexanone; 1,3-ditertbutylbenzene and malonic acid, hexyl 3-methylbutyl ester. Our results thus suggest the possible existence of generic VOC markers of pyrethroids in this strain. Of particular interest, two out of these three VOCs, the 1,3-ditertbutylbenzene and the malonic acid, hexyl 3-methylbutyl ester were found also in *B. megaterium* Mes11 when exposed with cypermethrine. This result highlighted the possible existence of interspecific VOC markers of pyrethroid in these two bacteria. Altogether, our work underlined the relevance of volatolomic to detect signatures of pesticides exposure in microorganisms and more generally to microbial ecotoxicology. Based on these first results, considerations of volatolomics for the chemical risk assessment in environment such as soils can be indirectly explored in longer terms.

## Introduction

In response to climatic or chemical stressors, organisms can trigger a set of metabolic adjustments to challenge cell damages and homeostasis disturbances (Bijlsma and Loeschcke, 2005; Hidalgo et al., 2016, 2018), resulting notably in the production of metabolic end-products like VOCs. VOCs are low molecular weight compounds with relatively high vapor pressure or volatility (Hakim et al., 2012). There is evidence that VOCs can be emitted from cells and their microenvironments in response to stressor perception, and can be used as indirect markers of contamination. Since the past decade, research in cancerology highlighted the interest of VOCs as specific markers to detect lung cancers and their distinct stages of maturity in humans (Hakim et al., 2012; Broza et al., 2015 for reviews). Briefly, several cytochrome p450 mixed oxidases are activated by exposure to environmental toxins. Such an enzyme system activation, linked with oxidative stress (e.g., reactive oxygen species production, lipid peroxidation, etc.), specifically modulates the catabolism of endogenous VOC products and generate an altered pattern of human breath by producing new VOCs or changing the ratio between VOCs that are normally produced in healthy bodies (Preti et al., 1988; Hakim et al., 2012). Volatolomics – the study of VOCs emitted by cells and their microenvironments – was also adapted in a context of food safety to diagnose xenobiotic contaminations in livestock (Berge et al., 2011; Bouhlel et al., 2017; Ratel et al., 2017). These works showed the relevance to study the liver volatolome of livestock species, like poultry, to reveal exposure to different micropollutants and pesticides. According to Bouhlel et al. (2017) and Ratel et al. (2017), VOCs determined as markers of contamination belong to different chemical families, including alkanes, alkenes, alcohols, and carboxylic acids. It is noteworthy that the type and the amount of VOCs differed according to the nature of the stressor analyzed. Finally, VOCs produced by microorganisms in response to chemical exposure also showed a recent increasing attention, because of possible medicinal, and environmental applications (Korpi et al., 2009; Scott-Thomas et al., 2013; Defois et al., 2017, 2018). For instance, Defois et al. (2017) underlined the relevance of using volatolomics to diagnose the exposure to benzo[*a*]pyrene and its associated metabolic deviations in the human gut microbiota. Accordingly, many VOCs are pointed out as markers of the gut microbiota exposure to benzo[*a*]pyrene, enlarging the potential applications of volatolomics in microbial ecotoxicology studies. To date, the use of volatolomic in microbial and/or bacterial ecotoxicology was restricted to community levels (Defois et al., 2017, 2018), but it is also of importance to investigate how chemical stressors in single populations influence VOCs emissions. Such an approach will be complementary and pioneer in the field of omic and microbial ecotoxicology. In time, dealing with bacteria, it is recommended to challenge different levels of biological organizations. Indeed, metabolic adaptation in response to stressors can be quickly selected allowing bacteria to adapt themselves to habitat fluctuations. In addition, responses of single populations under controlled conditions copy individual responses. Overall, this will help to understand mechanisms involved by bacteria to challenge and survive a chemical exposure by checking the VOCs that represents markers of metabolic deviation.

Volatolomics may thus offer a promising prospect to diagnose specific metabolic signatures and associate response of bacteria to chemical stressors like pesticides. Pesticides are of great concern as environmental pollutants resulting in human and animal health implications and many environmental side effects (Aktar et al., 2009; Parrón et al., 2014; Tsaboula et al., 2016). Although conventional targeted analytical methods used to monitor the pesticide levels are efficient, the implementation of less expensive and more straightforward analytical approaches could allow the existing surveillance system to be strengthened (Meurillon et al., 2017). In addition, available tools used to evaluate the ecotoxicological impact of pesticides in fields and organisms are very few, and there is a strong need of new approaches to enable a better understanding and prediction of effects as well as a deeper knowledge of the action modes of pesticides.

Soils are mainly contaminating by pesticide due to agricultural pratices. Therefore, in the present work, we designed an experimental volatolomic test devoted to identify indirect VOC markers of exposure of two soil bacterial strains to three different pesticides (deltamethrine, cypermethrine and sulcotrione). We choose to work with *Pseudomonas fluorescens* strain SG-1 and *Bacillus megaterium* strain Mes11, two common soil strains characterized based on works on pesticide degradations and selected from enrichment culture conditions (Batisson et al., 2009; Bardot et al., 2015; Carles et al., 2016). Based on previous works dealing with the exposure of bacteria communities and other organisms to chemical contaminants (Berge et al., 2011; Bouhlel et al., 2017; Defois et al., 2017), we assumed that exposure to pesticides should influence the metabolic pathways of our two bacteria strains, resulting in specific changes in their volatolome. We also hypothesized that changes in the bacteria volatolome should depends on the nature of the pesticide used and should differed between the two strains. To test these assumptions, a volatolomic pipeline was optimized from previous works devoted on livestock and microbial communities and adapted to single bacterial population study. Accordingly, the volatolome variations and/or deviations of the two bacteria strains exposed or not to two common landscape pyrethroid insecticides, the deltamethrine and the cypermethrine, and a triketone herbicide, the sulcotrione, were investigated and compared. In order to highlight candidate VOC markers associate with metabolic deviations of bacteria exposure to pesticides and not with altered phenotype experiments were conducted with pesticides at an EC10 (effective concentration that decrease the generation time of 10 %) concentration.

## Materials and Methods

### Bacterial Strains and Culture Conditions

Two edaphic bacterial strains isolated from soils from enrichment culture, *P. fluorescens* SG-1 (Carles et al., 2017) and *B. megaterium* Mes11 were used in this study. Both strains were maintained in laboratory in petri dishes on a Tryptic Soy Broth medium formulation (Sigma Aldrich, France). For experiments, strains were grown in 30 mL of medium growth solution (5 g/l peptone, 2.5 g/l yeast extract, 1 g/l D-Glucose) maintained in dark condition at 28°C under orbital agitation (200 rpm). To ensure enough bacterial biomass for further experiments, medium inoculation consist about 2 × 10e^4^ cells/ml and 2.5 × 10e^6^ cells/ml for *B. megaterium* Mes11 and *P. fluorescens* SG-1, respectively. *Pseudomonas fluorescens* SG-1 was pre-cultivated under these conditions overnight 12 h, whereas *B. megaterium* Mes11 was pre-cultivated for 48 h.

### Pesticide Exposure

**FIGURE 1:**
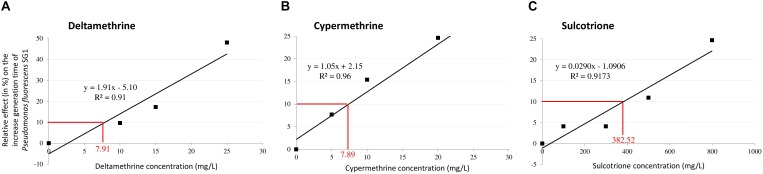
Relative effect of Deltamethrine **(A)**, Cypermethrine **(B)** and Sulcotrione **(C)** concentrations on the generation time of *Pseudomonas fluorescens* SG-1. For each pesticide, a linear regression was plotted to estimate the effective concentration at 10% inhibition (EC10) in generation time.

**FIGURE 2:**
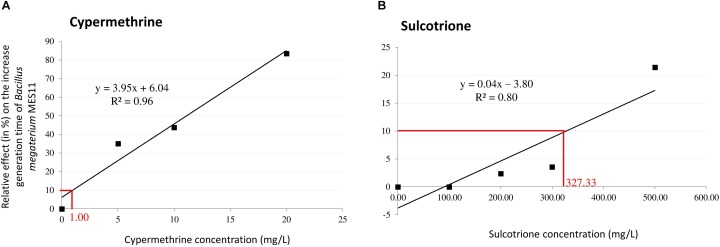
Relative effect of Cypermethrine **(A)** and Sulcotrione **(B)** concentrations on the generation time of *Bacillus megaterium* Mes11. For each pesticide, a linear regression was plotted to estimate the effective concentration at 10% inhibition (EC10) in generation time.

Deltamethrine, cypermethrine and sulcotrione were purchased from Sigma (Fluka, PESTANAL^®^, analytical standard). DMSO was used at a final concentration of 0.5% to enhance solubility of the three pesticides. The effective concentration that decrease the generation time of 10 % (EC10) was determined for each pesticide in both strains. Indeed, to guarantee a situation where deviation of the metabolome is linked to the presence of toxicant with phenotypic trait (Poynton and Vulpe, 2009), the experiments of pesticide exposure of bacteria were performed with a light toxic pressure (CE10 level). Using previous pre-cultured conditions for the two strains, we monitored every 30 min the bacterial biomass (OD 600 nm) among different pesticide concentrations ranging from 0 to 25 mg/L for deltamethrine and cypermethrine, and from 0 to 800 mg/L for sulcotrione. Accordingly, in *P. fluorescens* SG-1 EC10 were 7.91, 7.89, and 382.52 mg/L for deltamethrine, cypermethrine and sulcotrione, respectively (Figure 1). In *B. megaterium* Mes11, no reproducible EC10 could be determined for deltamethrine exposure, therefore we did not analyze this pesticide in this strain. However, the *B. megaterium* Mes11 EC10 were 1.00 and 327.33 mg/L for cypermethrine and sulcotrione, respectively (Figure 2).

For volatolomics study, 7–9 biological replicas for each pesticide and strain were prepared as already described, representing the “treated” samples. A 150 μL solution of pesticides solubilise in 0.5% of DMSO were added into a 30 mL Falcon containing 3 or 6 mL of *P. fluoresecens* or *B. megaterium*, respectively, in order to reach the EC10 concentration (determined in Figures 1, 2). Falcons were completed with growth medium until 30mL. Control samples contained only DMSO at a 0.5% final concentration. Experiments were conducted in Falcon tubes in 30 ml cultivation volume in dark condition at 28°C under orbital agitation (200 rpm) during 90 min. Bacterial biomass was monitored during experiments in order to check EC10. According to (Defois et al., 2017, 2018), we compared the relative abundance of VOCs from control samples (bacteria+DMSO) with treated ones (bacteria+DMSO+pesticide).

After 90 min of exposure, all samples were centrifuged during 7 min at 6000 rpm. Supernatant was removed and the bacteria were washed with a 0.8% NaCl solution and centrifuged again 7 min at 6000 rpm. This washing step was repeated twice. Bacteria were transferred into a cryo-tube directly plunged into liquid nitrogen to stop any metabolic activities. Samples were stocked at -80°C until further processing. This freezing process is crucial to stabilize VOCs for further analysis. Biological repetitions were settled from 7 to 9.

### Bacterial Volatolome Analyses

Volatile organic compounds (VOCs) of both *P. fluorescens* SG-1 and *B. megaterium* Mes11 were analyzed using a solid-phase microextraction (SPME) method coupled with gas chromatography-mass spectrometry (GC-MS) adapted from Bouhlel et al. (2017). Samples were progressively defrosted from -80°C to 4°C and maintained at 4°C until the end of experiments. For each sample, 250 μL of bacteria were transferred into a 10 mL vial supplemented with 700 μL of saturated saline solution at 360 g/L to facilitate VOC trapping as described in Bouhlel et al. (2017). Vial headspaces were set up under a nitrogen gas flow to avoid any oxygenation reactions. Vials were sealed and stored at 4°C during 24 h. Using an automated sampler (MPS2, Gerstel), samples were preheated in an agitator (500 rpm) for 10 min at 40°C, then the VOCs were trapped by SPME using a 75 μm carboxen-polydimethylsiloxane fiber (CAR/PDMS, 23gauge needle, Supelco) during 30 min at 40°C. The choice of the fiber and the analytical conditions were fixed by preliminary tests to limit thermal-induced VOC generation and to recover microbial VOCs with reduced variability. After a thermal desorption of the fiber at 280°C for 2 min in splitless mode in the GC inlet, the volatolome analysis was performed by GC-full scan MS (Shimadzu, QP2010+). VOCs were separated on a Rxi^®^-624Sil MS fused silica column (60 m × 0.25 mm × 1.4 μm, Restek) according to previously established settings (Bouhlel et al., 2017; Defois et al., 2017). By using the same extraction and separation parameters, pools of samples of each group of bacteria tested were analyzed by SPME-GC hyphenated with high resolution mass spectrometry (Q Exactive GC Orbitrap, Thermo Scientific). The analyses were performed both with chemical ionization (CI) and electron ionization (EI) in order to comfort VOC detection and enhance identification.

Peak areas of VOCs were determined using a mass fragment selected for its specificity and free from co-elution with an automatic algorithm developed in our laboratory under Matlab R2017 by Bouhlel et al. (2017). VOCs selected as discriminant for each pesticide exposure were tentatively identified according to a comparison between their mass spectra and the NIST 14 mass spectral library. All VOC identifications were validated through the SPME-GC-HRMS signals obtained in IC and IE modes.

Finally, the three pesticide solutions (deltamethrine, cypermethrine, sulcotrione) used to expose the bacteria were also analyzed at EC10 concentration by similar protocol as described for samples in order to verify if candidate VOCs markers of pesticide exposure had an exogenous origin.

### Statistical Analyses

Datasets were processed using the *R* 3.1.1 statistical software (R Development Core Team, 2008). First MANOVA analysis were assessed to test the significance of pesticide exposure of VOC patterns of both strains. Then, Student *t*-test adjusted by Bonferroni corrections were used to assess the significant influence of each pesticide exposure on the volatolome of both bacteria strains. For each strain and each pesticide, a principal component analysis (PCA) was performed based on discriminant VOCs selected by the ANOVAs to visualize the data structuration (Bouhlel et al., 2018).

## Results

### Exposure to Pesticides Influences the Volatolome of *Pseudomonas fluorescens* SG-1

The analytical pipeline identified a total of 134 VOCs in *P. fluorescens* SG-1. Results showed a significant differences of VOC patterns of strain exposed to pesticides compare to control ones (MANOVA, *ddl* = 1, *F*_Delta_ = 7.2; *F*_Cyper_ = 4.8; *F*_Sulcor_ = 8.4, *P*-value < 0.05). Accordingly, eight, six and three of them were over- or under-expressed following exposure to deltamethrine, cypermethrine and sulcotrione, respectively, meaning that pesticides are associated with a volatolome deviation in strains.

**FIGURE 3:**
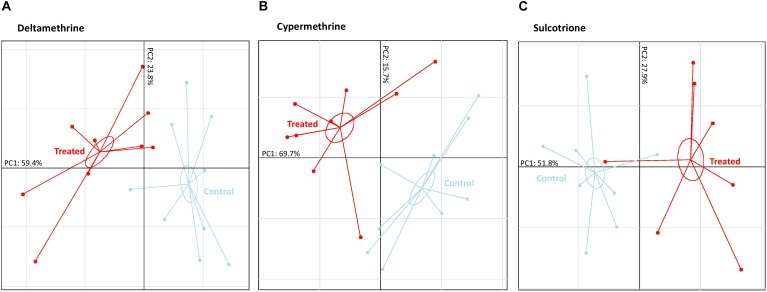
*Pseudomonas fluorescens* SG-1 volatolome patterns according they were exposed to three different pesticides (7 to 9 “Treated” samples represented in red) or not (8 to 9 “Control” samples represented in light blue). **(A)** Principal component analysis (PCA) based on the deltamethrine dataset. **(B)** Principal component analysis (PCA) based on the cypermethrine dataset. **(C)** Principal component analysis (PCA) based on the sulcotrione dataset. Each point represent a sample and inertia ellipse size were represented at a coefficient of 0.3.

Based on these discriminant VOCs, PCAs were performed for each pesticide to visualize the structure of data and which compounds were over- and/or down-expressed in treated specimens (Figure 3). The first PCAs axes (PC1), which explained the main dataset inertia distribution (51 to 69%), showed a very clear separation of “treated” samples with “control” ones. By contrast, PC2 (15.7–27.9% of the total dataset inertia) were built on the natural biological variation of *P. fluorescens* SG-1. Potential markers of *P. fluorescens* SG-1 exposure to pesticide were thus found among the most contributing VOCs to PC1.

When exposed to deltamethrine, PC1 separation was mainly due to the increase expression of carbonyl sulfide, two branched-alkanes (2,3,3-trimethylpentane and 2,3-dimethylhexane), malonic acid, hexyl 3-methylbutyl ester, and one aromatic compound (1,3-ditertbutylbenzene), and to the decrease expression of ketone (2-hexanone) and two others unidentified VOCs despite the high-resolution mass spectrum assay (Table 1).

**Table 1:** Significant volatile metabolites over- or down-expressed in the volatolome of *Pseudomonas fluorescens* SG-1 strain when exposed to deltamethrine, cypermethrine, or sulcotrione pesticides compared to control.

Pesticide	Volatile metabolite^1^	m/z	RT^2^	Mean peak abundance ± ER (×10^3^)		Statiscal *P*-value of student *t*-tests^3^
					
				Treated	Control	
**DELTAMETHRINE**	**Over-expressed in “treated” bacteria**					
	Carbonyl sulfide	60	4.6	50.8 ± 3.5	38.5 ± 1.9	<0.01
	2,3,3-Trimethylpentane	43	22.8	3056.0 ± 66.4	111.1 ± 30.6	<0.05
	2,3-Dimethylhexane	70	23.3	140.8 ± 27.9	53.9 ± 10.8	<0.05
	Malonic acid, hexyl 3-methylbutyl ester	71	38.3	35.9 ± 3.2	23.42 ± 2.3	<0.01
	1,3-Ditertbutylbenzene	57	53.2	166.3 ± 21.9	46.4 ± 12.0	<0.001
	**Down-expressed in “treated” bacteria**					
	2-Hexanone	58 and 43	27.5	42.5 ± 2.3	49.75 ± 2.5	<0.05
	Unidentified	57	25.8	18.8 ± 0.8	23.17 ± 2.0	<0.05
	Unidentified	71	72.3	25.1 ± 2.2	47.35 ± 5.3	<0.01
**CYPERMETHRINE**	**Over-expressed in “treated” bacteria**					
	2-Butanone	72	14.8	217.0 ± 17.3	159.1 ± 21.2	<0.05
	2-Methyl-2-butanol	59	17.5	197.1 ± 19.4	143.0 ± 12.7	<0.05
	2-Pentanone	43	20.6	52.3 ± 3.1	41.5 ± 4.1	<0.05
	2-Hexanone	58 and 43	27.5	54.6 ± 2.4	46.5 ± 2.7	<0.05
	Malonic acid, hexyl 3-methylbutyl ester	71	38.3	28.7 ± 1.1	23.2 ± 0.7	<0.001
	1,3-Ditertbutylbenzene	87	53.2	94.8 ± 8.0	36.2 ± 4.4	<0.001
**SULCOTRIONE**	**Over-expressed in “treated” bacteria**					
	2,3,3-Trimethylpentane	43	22.8	76.4 ± 7.2	54.6 ± 3.2	<0.05
	Toluene	91	25.1	307.6 ± 20.7	234.9 ± 21.3	<0.05
	**Down-expressed in “treated” bacteria**					
	Unidentified	43	24.1	24.3 ± 2.2	30.1 ± 1.5	<0.05


*VOC are expressed with an arbitrary unity of area.*

*^*1*^Tentative identification based on high resolution mass spectrum and EI/CI spectra from literature and internal databank.*

*^*2*^Retention time indices on a Rxi^®^-624Sil MS fused silica column (60 m × 0.25 mm × 1.4 μm, Restek).*

*^*3*^*P*-value resulting for *t*-test abundance differences between treated and control groups (*df* = 1).*

By comparison, when exposed to cypermethrine, bacteria exhibited a significant increase expression of three ketones (2-butanone, 2-pentanone, 2-hexanone), one alcohol (2-methyl-2-butanol), one acid (malonic acid), one ester (hexyl 3-methylbutyl ester) and one aromatic compound (1,3-ditertbutylbenzene), whereas no VOC reduction was observed (Table 1). When exposed to sulcotrione, bacteria exhibited a significant increase of one branched-alkane (2,3,3-trimethylpentane) and one aromatic compound (toluene), an exogenous benzenic compound being part of the sulcotrione volatolome, and decrease expression of one unidentified VOC (Table 1).

### Exposure to Pesticides Influences the Volatolome of *Bacillus megaterium* Mes11

**FIGURE 4:**
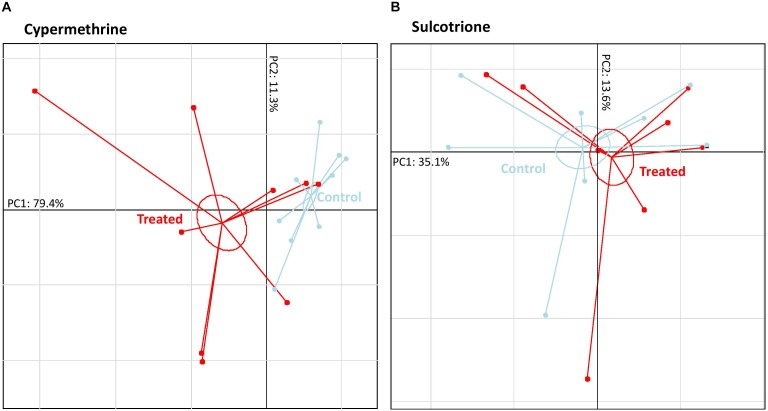
*Bacillus megaterium* Mes11 volatolome patterns according they were exposed to three different pesticides (8 to 9 “Treated” samples represented in red) or not (8 to 9 “Control” samples represented in light blue). **(A)** Principal component analysis (PCA) based on the cypermethrine dataset. **(B)** Principal component analysis (PCA) based on the sulcotrione (no groups separation) dataset. Each point represent a sample and inertia ellipse size were represented at a coefficient of 0.3.

As shown in Figure 4 *B. megaterium* Mes11 only showed a significant alteration of its volatolome when exposed to cypermethrine. Results showed a significant differences of VOC patterns of strain exposed to pesticides compare to control ones (MANOVA, *ddl* = 1, *F*_Cyper_ = 5.7; *F*_Sulcor_ = 8.9, *P*-value < 0.05). Out of 150 VOCs which were identified in *B. megaterium* Mes11 volatolome, 15 were over or under-expressed following cypermethrine exposure. All of them seem to be endogenous to *B. megaterium* Mes11 (no one were identified in the cypermethrine specific volatolome).

The clear case-control separation exhibited by PC1 (79.4%) was explained by the increase expression of five branched-alkanes (2,3,3-trimethylpentane, 2,3-dimethylhexane, 2,2,4-trimethylhexane, 2,6-dimethylnonane, 3-ethylpentane), one alkane diol (3-methoxy-hexane-1,6-diol), one branched-alkene (2,4-dimethyl-1-heptene), one acid (malonic acid), one ester (hexyl 3-methylbutyl ester), one alcohol (2-hexyl-1-octanol), one aromatic compound (1,3-ditertbutylbenzene) and five unidentified VOCs (Table 2).

**Table 2:** Significant volatile metabolites over-expressed in the volatolome of *Bacillus megaterium* Mes11 strain when exposed to cypermethrine pesticides compared to control.

Volatile metabolite^1^	m/z	RT^2^	Mean peak abundance ± ER (x10^3^)		Statiscal *P*-value of student *t*-tests^3^
				
			Treated	Control	
**Over-expressed in “treated” bacteria**					
3-Ethylpentane	43	22.3	324.4 ± 60.7	131.7 ± 31.9	<0.05
2,3,3-Trimethylpentane	43	22.8	518.0 ± 107.4	204.2 ± 53.3	<0.05
2,3-Dimethylhexane	70	23.2	128.3 ± 23.0	53.7 ± 11.5	<0.05
2,2,4-Trimethylhexane	57	24.2	413.3 ± 82.9	178.3 ± 52.5	<0.05
2,4-Dimethyl-1-heptene	43	28.5	185.8 ± 33.0	70.9 ± 15.2	<0.01
2,6-Dimethylnonane	57	38.9	90.4 ± 22.2	41.4 ± 450	<0.05
Malonic acid, hexyl 3-methylbutyl ester	43	38.9	130.1 ± 32.7	60.5 ± 8.1	<0.05
3-Methoxy-hexane-1,6-diol	43	41.0	19.6 ± 1.6	14.3 ± 1.2	<0.05
2-Hexyl-1-octanol	43	43.7	26.5 ± 5.2	14.0 ± 0.9	<0.05
1,3-Ditertbutylbenzene	57	53.2	241.5 ± 61.8	73.5 ± 8.4	<0.05
Unidentified	55	28.9	45.6 ± 5.4	25.2 ± 3.7	<0.01
Unidentified	57	32.0	157.4 ± 39.1	67.7 ± 10.4	<0.05
Unidentified	57	32.3	45.0 ± 11.0	22.0 ± 2.9	<0.05
Unidentified	57	32.4	77.4 ± 20.7	32.6 ± 4.3	<0.05
Unidentified	71	33.4	44.1 ± 9.9	21.8 ± 2.2	<0.05


*VOC are expressed with an arbitrary unity of area.*

*^*1*^Tentative identification based on high resolution mass spectrum and EI/CI spectra from literature and internal databank.*

*^*2*^Retention time indices on a Rxi^®^-624Sil MS fused silica column (60 m × 0.25 mm × 1.4 μm, Restek).*

*^*3*^*P*-value resulting for *t*-test abundance differences between treated and control groups (*df* = 1).*

As showed in *P. fluorescens* SG-1, PC2 was built on the natural biological variation of bacteria.

## Discussion

The present work aimed at identifying metabolic VOC markers of exposure to three different pesticides used at low concentration (EC10) in two edaphic bacteria strains, *P. fluorescens* SG-1 and *B. megaterium* Mes11. Using a volatolomic approach, we first highlighted a set of bacterial VOCs that seem to be specifically modulated in each strain according to the nature of the pesticide used. Second, our results revealed three VOCs (1,3-Ditertbutylbenzene, 2-Hexanone and Malonic acid, hexyl 3-methylbutyl ester) modulated in *P. fluorescens* SG-1 when exposed with pyrethroid pesticides (deltamethrine and cypermethrine), suggesting the existence of candidate generic VOC markers of pyrethroids in this strain. Interestingly, two out of these three VOCs were found in *B. megaterium* Mes11 when exposed with cypermethrine too, suggesting the existence of interspecific markers of pyrethroid in these bacteria. Altogether, our results underlined the relevance of volatolomics to detect signatures of pesticide exposure in microorganisms.

### Bacterial VOCs Can Be Robust and Specific Strain Markers of Different Pesticide Exposure

The volatolomic approach revealed several markers of pesticide exposure in both strains. This is particularly observed in *P. fluorescens* SG-1. Bacteria modulated a set of strain-specific VOCs following exposure to deltamethrine, cypermethrine or sulcotrione. Accordingly, when they were exposed to deltamethrine, *P. fluorescens* SG-1 showed higher levels in carbonyl sulfide and two branched-alkanes (2,3,3-trimethylpentane and 2,3-dimethylhexane). The increased levels of these three VOCs should be the results of distinct metabolic responses displayed by the strain to challenge the impacts of deltamethrine in cells. According to previous works, the release of carbonyl sulfide could be linked with an increase of metabolic rate and respiration in plants and some soil microorganisms (Campbell et al., 2008; Maseyk et al., 2014). Here the increase of carbonyl sulfide by *P. fluorescens* SG-1 could reflect a rise of metabolic rate in cells because of larger demands of energetic substrates (i.e., NADPH, ATP) to counteract the impacts of deltamethrine at cell levels. Indeed, there is many evidence that species over-produce such an energetic substrate when they are exposed to environmental stressful conditions (Hidalgo et al., 2014). NADPH and ATP are non-negligible fuel for metabolic pathways of cell detoxifications and cell membrane protections. Strengthening this hypothesis, there were also evidences that carbonyl sulfide is released in the headspace of bacteria like *Thiobacillus thioparus* THI115 and *Pseudomonas aeruginosa STK 03* during thiocyanate degradation (Kim and Katayama, 2000; Mekuto et al., 2016), a process requiring a non-negligible quantity of energetic substrates. The two branched-alkanes 2,3,3-trimethylpentane and 2,4-dimethylhexane had been already proposed as candidate markers of human lung tumors (Filipiak et al., 2008, 2010, 2016), and their over-expression is linked to the peroxidation of phospholipid membranes. In rats the evidence that exposure to deltamethrine is showed to increase lipid peroxidation (Nasuti et al., 2003). Such a lipid peroxidation can thus occurred in our bacteria conducing to the over-expression of the two branched-alkane VOCs.

By contrast, when they were exposed to cypermethrine, *P. fluorescens* SG-1 showed the over-production of 2-methyl-2-butanol and two ketones, 2-butanone and 2-pentanone. There were evidence that these last two ketones can be used as relevant tumor-markers in human (Filipiak et al., 2008). In bacteria, both 2-butanone and 2-pentanone are generated by lipolysis processes (Vallerand et al., 1999; Zhang et al., 2012).

Finally, when they were exposed to sulcotrione, *P. fluorescens* SG-1 showed the over-production of two compounds, 2,3,3-trimethylpentane and toluene. We already discussed the increase level of 2,3,3-trimethylpentane under deltamethrine exposure, suggesting lipid peroxidation might also take place in *P. fluorescens* under sulcotrione exposure. Regarding toluene, the release of this VOC in exposed strain should be contrasted in regards with a possible exogenous origin. We showed (Supplementary Data 1) that this aromatic VOC is found in the volatolome of the sulcotrione. Indeed, toluene is well-known by-product of the manufacturing process of sulcotrione.

In *B. megaterium* Mes11, the volatolome was also modulated, notably when the strain was exposed to cypermethrine. Fifteen discriminant VOCs were over-expressed. Eight were found only in *B. megaterium* Mes11 and were correctly identified. We found six branched-alkanes (3-ethylpentane, 2,3,3-trimethylpentane, 2,3-dimethylhexane, 2,2,4-trimethylhexane, 2,6-dimethylnonane and 3-methoxy-hexane-1,6-diol), one branched-alkene (2,4-dimethyl-1-heptene), and one primary alcohol (2-hexyl-1-octanol). Exposure to pyrethroids induced many modifications at cellular levels, including lipid peroxidation of fatty acids that form part of the cell membranes, and the synthesis of polyunsaturated fatty acids (PUFAs). In response, organisms display number of metabolites from oxidation reactions catalyzed by enzyme systems associated with cytochrome p450 and alcohol dehydrogenases (ADH). These metabolites include alkanes (C3-C11), branched-alkanes and -alkenes, alcohols and also aldehydes and carboxylic acids as end metabolites of the reaction (Hakim et al., 2012; Jareño-Esteban et al., 2013), same as showed in *B. megaterium*. Such VOCs markers might result from a complex equilibrium between non-specific oxidative stress induced and probably more specific enzymatic detoxification activities whose induction could be more pesticide dependent. More interesting, the two branched-alkanes, 2,3,3-trimethylpentane and 2,3-dimethylhexane, were also over-expressed too in *P. fluorescens* when exposed to deltamethrine and sulcotrione. This result suggests that these two VOCs could be more global markers of oxidative stress in bacteria than specific markers of pesticide exposure. According to this hypothesis, there are evidence that these two branched-alkanes are over-expressed in species and humans during the development of cancerous tumors (Filipiak et al., 2008, 2010, 2016).

Altogether, our results suggest thus that volatolome of bacteria can be modulated in response to chemical stressors, and that this modulation might depend on both the strain and the stressor studied. Although experimental validation still required, monitoring bacterial VOCs could pave the way for future diagnosis methods of pesticide contamination in soil microorganisms.

### Bacterial VOCs Can Be Generic and Interspecific Strain Markers of Global Pyrethroid Exposure

Deltamethrine and cypermethrine are two pesticides belonging to the pyrethroid family. Exposure to these two pesticides should thus induce similar cell damages (i.e., pyrethroids act like axonic excitoxins preventing the closure of the voltage-gated sodium channels) and thus physiological modulations in organisms, including volatolome adjustments. Corroborating this hypothesis, our approach has detected modulated expression of three VOCS (2-hexanone, 1,3-ditertbutylbenzene and malonic acid, hexyl 3-methylbutyl ester) in *P. fluorescens* strain SG-1 when exposed to deltamethrine and cypermethrine. First, a higher expression levels of 1,3-ditertbutylbenzene and malonic acid, hexyl 3-methylbutyl ester under both pesticide exposure. However, the expression modulation of 2-hexanone is interesting, as there is evidence in arthropods that the VOC can have an inhibitory effect on voltage-gated sodium channels (Papachristoforou et al., 2012), the activities of which are altered by pyrethroids exposure. Interestingly, our results showed that the modulation level of 2-hexanone in *P. fluorescens* depends on the nature of the pyrethroid the strain is exposed. The VOC increased when strain was exposed to cypermethrine, while it decreased when exposed to deltamethrine. In some microbial studies, an increased level of 2-hexanone was detected before remedial actions, whereas it decreased after mitigations, conducing to controversial results (Korpi et al., 2009). Further works are required to test it, but the contrasting modulations of 2-hexanone in *P. fluorescens* could suggest that strain had already started or finished the mitigation of deltamethrine 90 min after exposure while it did not started yet with cypermethrine.

Regarding the levels of two VOCs (1,3-ditertbutylbenzene and malonic acid, hexyl 3-methylbutyl ester), an increase was observed in *P. fluorescens* SG-1 under pyrethroid (deltamethrine and cypermethrine) exposure, and in *B. megaterium* Mes11 under cypermethrine exposure. Although we have no data about how deltamethrine influences the volatolome of *B. megaterium*, the two VOCs seem to be interspecific markers of cypermethrine contamination in the two strains. Based on the detection of such generic and interspecific markers of cypermethrine exposure, we could further envisage risk assessment investigations in more complex ecosystemic fields like soil mesocosms. However, more studies are still required in order to comfort an interpretative marker of these two compounds. Investigating physiological incidences of these two VOCs on bacteria may help to clarify the origin of these compounds as candidate markers of many mammal diseases related to lipid peroxidation processes (Hanai et al., 2012; Filipiak et al., 2016; Tang et al., 2017).

### Can *Bacillus megaterium* Mes11 Dissipate the Sulcotrione?

The PCA analysis did not reveal significant volatolome difference in *B. megaterium* Mes11 whether they were exposed to the sulcotrione or not. *B. megaterium* Mes11 was characterized as an efficient mesotrione-degrading strain (Batisson et al., 2009; Bardot et al., 2015; Carles et al., 2016), a selective herbicide belonging to the triketone family like the sulcotrione. Briefly, *B. megaterium* Mes11 isolated from soils was able to completely and very rapidly (about 1 h at 30 mg/L) biotransform the mesotrione into 2-amino-4methylsulfonylbenzoic acid (AMBA) and 4-methylsufonyl-2-nitrobenzoic acid (MNBA) in its culture growth (Batisson et al., 2009; Carles et al., 2016) by nitro-reduction and hydrolysis. In order to dissipate the sulcotrione from its medium growth, a first hypothesis could be that bacteria degrade the pesticide. Indeed, many microorganisms including *Tetrahymena pyriformis* and *Vibrio fischeri* were shown to be able to degrade both sulcotrione and mesotrione herbicides. Although we cannot rule out the hypothesis that the vehicle (DMSO 0.5%) might hide the effects of sulcotrione on the strain volatolome, we can first supposed that *B. megaterium* Mes11 should rapidly degrade the sulcotrione. However, such a sulcotrione-degrading activity of *B. megaterium* Mes11 have to be further investigated by measuring the sulcotrione and its residues directly in strain cells and growth medium along the experiment duration.

Second, triketone pesticides can have genotoxic effects on cells, as already demonstrated in plants (Dumas et al., 2017). Therefore, the exposure to 327.33 mg/L of sulcotrione in *B. megaterium* Mes11 could induce a 10% decline of their generation time because of a genotoxic effect on cells (impacting cellular division) rather than detoxification processes at the metabolic scale. Similar conclusions had been made in the protist *Paramecium* in which exposure to sulcotrione influences the transcription of genes involved in cell division whereas exposure to deltamethrine influences the transcription of detoxifying genes (Bouchard et al. Not published yet).

Third, *Bacillus* can turned to resistance form by encapsulation when they were exposed to chemical stress. The decline of generation time measured with 327.33 mg/L of sulcotrione could also be the consequence of such a protective encapsulation state of the strain rather than a consequence of stress detoxification.

### Reinvest the Concept of Bacterial Ecotoxicogenomic Thanks to Volatolomic

Conventional analyses used to detect and quantify the presence of xenobiotics in the environment provide an efficient way to improve risk assessments, but do not estimate how they influence biological systems inhabiting these ecosystems. It is in this context that ecotoxicogenomics, a set of analytical tools that combines high throughput DNA technologies with bioinformatics, had emerged (Hamadeh et al., 2002; Robbens et al., 2007; Afshari et al., 2011). Thus, rather than determining whether a chemical compound is toxic, ecotoxicogenomics proposed to determine their mechanisms of action in organisms. However, chemical contaminants have not one but rather a series of molecular effects that can vary in time and with concentration. There are genes responding within a short period of time (hours), others that are only differentially regulated after longer terms of exposure, or transiently expressed. Some genes will probably be more sensitive than others, so exposure to low concentrations of contaminants may affect a reduced suite of genes, while at higher concentrations, a much larger suite of genes may well be affected. Very high concentrations of a chemical may be toxic and lead to other genes being affected because of the cellular damage. Metabolomics and more particularly volatolomics, which is interested in the volatile fraction of the volatolome, captures a more integrated assessment of the physiological state of an organism that transcriptomics and proteomics do (Ankley et al., 2006; Fent and Sumpter, 2011). Indeed, volatile compounds are the terminal products of a toxic response. Their production integrates gene transcription alteration and the reorientation of stress metabolism. It stacked the whole cell damage as a simple under or overproduction of short, low mass, volatile molecule. We can mind in term of production and not expression. Patterns in of volatile profiles may thus offer the potential to uncover novel markers of exposure to chemical contaminants in organisms or ecosystems. In addition, this can be achieved at very low concentration. For instance, the No Observed Transcriptional Effect Level (NOTEL) may play a role in determining if a predicted environmental concentration poses a risk to a sensitive organism within an ecosystem. The pollutant interacts with cells and cellular components in a manner dependent on its chemical properties resulting in specific cellular damage or stress responses (Poynton and Vulpe, 2009). Gene expression might not be affected at the NOTEL concentration, but the cell detoxication pathways are yet producing volatile compounds that are the consequence of a stress response.

## Conclusion

In our knowledge, no previous work had already used volatolomics to investigate indirect markers related to the exposure of soil bacterial strains to different pesticides. We thus optimized a volatolomic pipeline from previous works devoted to livestock and microbial communities (Bouhlel et al., 2017; Defois et al., 2017; Ratel et al., 2017) and adapted it to single bacterial population study. Here, we demonstrated that a short-time exposure (90 min) to different pesticide molecules, modulated a set of potentially strain-specific VOCs in bacteria, the nature of which depends on metabolic and enzymatic adjustments displayed to counteract the impacts of pesticides in cells. Such VOCs are probably markers of pesticide exposure. By increasing the time of exposure, it will be of interest to re-start analyses in order to study the mechanism of action of the pesticides and their cellular resilient responses. Of particular important is the potential existence of two VOCs that could be proposed as candidate interspecific markers of pyrethroid pesticide family in bacteria. More studies still required comforting this hypothesis, but these results should pave the way to alternative analytical set-up in microbial ecotoxicology. For a further chemical risk assessment in real environmental matrices based on volatolomics, VOC pattern recognition should be studied according to bacteria strain, pesticide concentration and exposure time. One first solution could be to target volatolome in simplified soil microcosm (2–3 strains). To date, one of the main bottleneck to engage such of microcosm study will be to isolate a sufficient concentration of bacteria without damaging them in order to prevent the mix of VOCs in the microcosm and get a measurable and interpretable signal. We consider that combine volatolomics with other omic tools (transcriptomics and proteomics) should enlarge further diagnosis applications on real biodiversity soils.

## Author Contributions

This work was driven by EE and PB. EE, PB, and KH designed and wrote the manuscript. KH conducted all experiments. FM and JR helped KH in adapting the volatolomic assay for single bacteria. BG conducted the HR-MS analysis to validate VOC identification.

## Conflict of Interest Statement

The authors declare that the research was conducted in the absence of any commercial or financial relationships that could be construed as a potential conflict of interest.

## Abbreviations

PCA: principal component analysis
VOCs: volatile organic compounds
