# Supplementary figures and images for: Volatolomics in Bacterial Ecotoxicology, A Novel Method for Detecting Signatures of Pesticide Exposure?
Source: Front Microbiol. 2019 Jan 8;9:3113. doi: 10.3389/fmicb.2018.03113 (PMC6332697; doi:10.3389/fmicb.2018.03113)

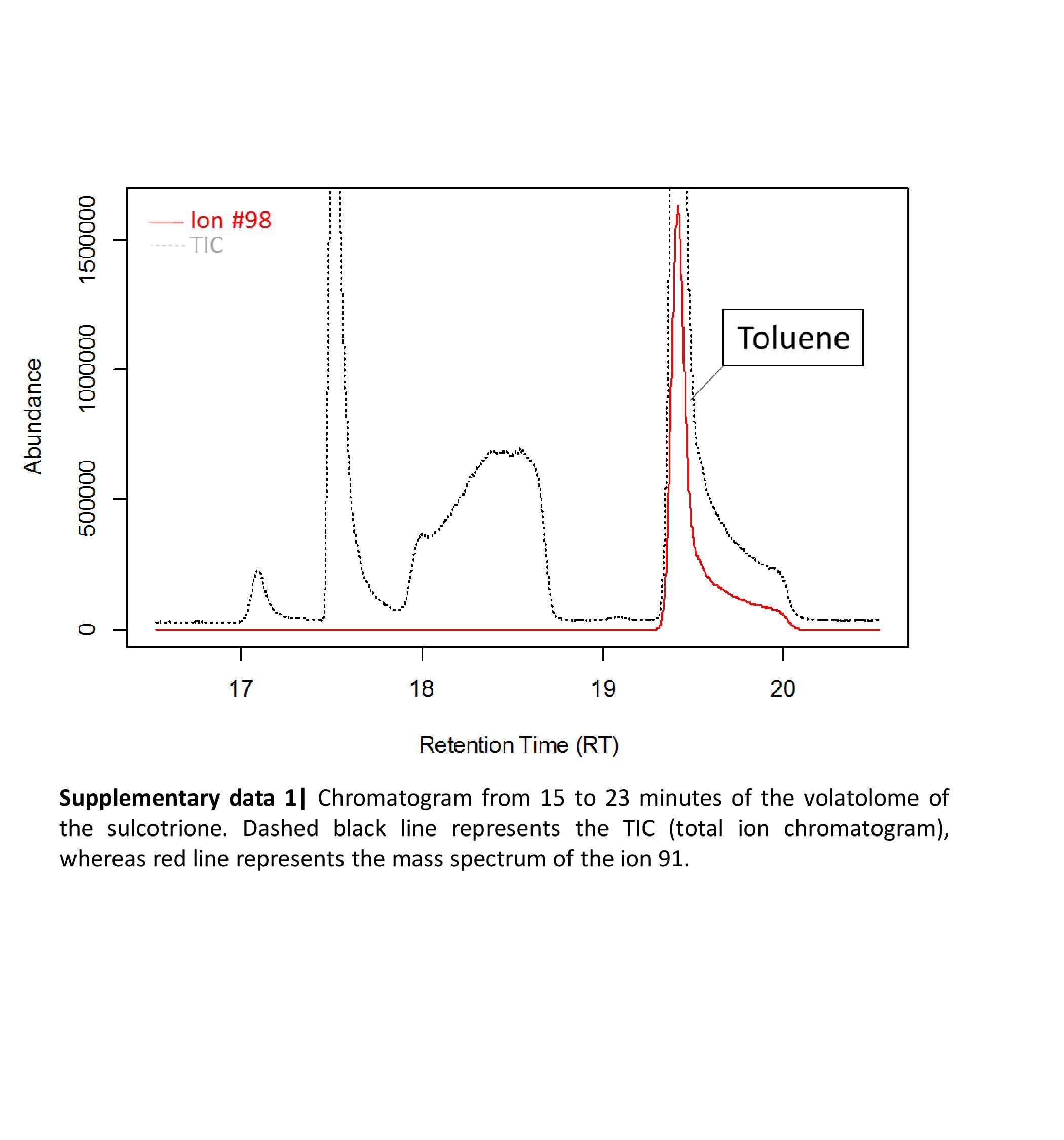

Supplement: Supplementary file 1 [file Image_1.JPEG]
